# Gender and Socioeconomic Inequality in the Prescription of Direct Oral Anticoagulants in Patients with Non-Valvular Atrial Fibrillation in Primary Care in Catalonia (Fantas-TIC Study)

**DOI:** 10.3390/ijerph182010993

**Published:** 2021-10-19

**Authors:** Mª Rosa Dalmau Llorca, Carina Aguilar Martín, Noèlia Carrasco-Querol, Zojaina Hernández Rojas, Emma Forcadell Drago, Dolores Rodríguez Cumplido, Elisabet Castro Blanco, Josep Mª Pepió Vilaubí, Alessandra Queiroga Gonçalves, José Fernández-Sáez

**Affiliations:** 1 Equip d’Atenció Primària Terres de l’Ebre, Institut Català de la Salut, 43500 Tortosa, Catalonia, Spain; rdalmau.ebre.ics@gencat.cat (M.R.D.L.); zojahernandez@gmail.com (Z.H.R.); eforcadellg.ebre.ics@gencat.cat (E.F.D.); 2 Campus Terres de l’Ebre, Universitat Rovira i Virgili, 43500 Tortosa, Catalonia, Spain; elicasblan@gmail.com (E.C.B.); jfernandez@idiapjgol.info (J.F.-S.); 3 Primary Care Intervention Evaluation Research Group (GAVINA Research Group), IDIAPJGol Terres de l’Ebre, 43500 Tortosa, Catalonia, Spain; caguilar.ebre.ics@gencat.cat (C.A.M.); drficf@gmail.com (D.R.C.); josepmp54@gmail.com (J.M.P.V.); 4 Terres de l’Ebre Research Support Unit, Foundation University Institute for Primary Health Care Research Jordi Gol i Gurina (IDIAPJGol), 43500 Tortosa, Catalonia, Spain; 5 Unitat d’Avaluació, Direcció d’Atenció Primària Terres de l’Ebre, Institut Català de la Salut, 43500 Tortosa, Catalonia, Spain; 6 Unitat de Recerca, Gerència Territorial Terres de l’Ebre, Institut Català de la Salut, 43500 Tortosa, Catalonia, Spain; 7 Hospital Universitari de Bellvitge, Institut Català de la Salut, 08907 Barcelona, Catalonia, Spain; 8 Unitat Docent de Medicina de Familia i Comunitària, Tortosa-Terres de l’Ebre, Institut Català de la Salut, 43500 Tortosa, Catalonia, Spain

**Keywords:** direct oral anticoagulants, vitamin K antagonists, atrial fibrillation, socioeconomic inequalities, sex-related differences

## Abstract

Background: Evidence points to unequal access to direct oral anticoagulant (DOAC) therapy, to the detriment of the most socioeconomically disadvantaged patients in different geographic areas; however, few studies have focused on people with atrial fibrillation. This study aimed to assess gender-based and socioeconomic differences in the prescriptions of anticoagulants in people with non-valvular atrial fibrillation who attended Primary Care. Method: A cross-sectional study with real-world data from patients treated in Primary Care in Catalonia (Spain). Data were obtained from the SIDIAP database, covering 287 Primary Care centers in 2018. Results were presented as descriptive statistics and odds ratios estimated by multivariable logistic regression. Results: A total of 60,978 patients on anticoagulants for non-valvular atrial fibrillation were identified: 41,430 (68%) were taking vitamin K antagonists and 19,548 (32%), DOACs. Women had higher odds of treatment with DOAC (adjusted odds ratio [ORadj] 1.12), while lower DOAC prescription rates affected patients from Primary Care centers located in high-deprivation urban centers (ORadj 0.58) and rural areas (ORadj 0.34). Conclusions: DOAC prescription patterns differ by population. Women are more likely to receive it than men, while people living in rural areas and deprived urban areas are less likely to receive this therapy. Following clinical management guidelines could help to minimize the inequality.

## 1. Introduction

### Gender-Based and Socioeconomic Differences in Health Care and Drug Prescription

Oral anticoagulants are the drugs of choice to prevent stroke in people with atrial fibrillation. In non-valvular atrial fibrillation, two classes of oral anticoagulants are available for preventing a thromboembolic event: vitamin K antagonists (VKA) and direct oral anticoagulants (DOAC). VKAs are characterized by a narrow therapeutic window, require frequent follow-ups, are effective for preventing stroke, increase the risk of bleeding, can be used in people with any degree of renal insufficiency, and are less expensive than DOACs. For their part, DOACs do not require monitoring, effectively prevent stroke, and also increase the risk of bleeding, although to a lesser extent than VKAs for intracranial bleeding [1,2].

The cost and follow-up profile of a given treatment can influence its prescription differently depending on certain factors and contexts. For example, prescription patterns can be conditioned by sociodemographic and economic factors that are unrelated to medication appropriateness criteria [3].

Different authors have analyzed the influence of deprivation in health inequalities, at both a national and international level [4,5,6,7]. Deprivation has been conceptualized since the 1970s [4], differing from the classical concept of poverty in that it is linked to difficulties (capability) in access to employment, education, culture and social development at levels considered acceptable for society. The concept of deprivation thus encompasses more than food insecurity, lack of basic goods such as clothing, sub-standard housing and other purely economic or monetary indicators of well-being, in consonance with a holistic model of health [8]. In Spain, several studies have applied deprivation indexes to different settings based on the MEDEA project (“Mortality and socioeconomic and environmental inequalities in small Spanish areas”) [8]. In England, socioeconomic deprivation was associated with opioid and non-opioid analgesics, antipsychotics and reflux medication prescriptions, while affluence was associated with epinephrine, combined oral contraceptives and hormone replacement therapy [9]. A recent meta-analysis showed that the rate of prescription of guideline-recommended medications in managing acute coronary syndrome was significantly different between patients with the lowest and the highest socioeconomic status [10]. Regarding oral anticoagulants, in Sweden, differences by age, income, education and country of birth were found in their prescribing after stroke. Those differences were not explained by common risk factors, indicating socioeconomic inequalities in the prescribing of preventive treatment after stroke [11]. In Denmark, patients with atrial fibrillation who had a low income, low education and were living alone were associated with a lower chance of being initiated with oral anticoagulation therapy, and new high-cost drugs were increasing inequality [12]. To date, few studies have assessed inequalities in the prescription of DOACs, which are more expensive than VKA, in patients with atrial fibrillation [6,13]. There are those that have highlighted the substantial disparities that exist around access to new anticoagulant therapies in the USA among socioeconomically disadvantaged patients and the need to study inequalities related to the prescription of oral anticoagulants [6,13]. Differences in DOAC prescription patterns have already been observed in relation to socioeconomic indicators [6]. Yet, any analysis of socioeconomic determinants must also take into account the gender dimension, as this is the relational aspect that governs how sex interacts with the world around it [14,15]. There is evidence that oral anticoagulants are prescribed less frequently to women compared to men with atrial fibrillation [16]; although, few studies have evaluated the variety of anticoagulant prescribed by gender [13]. Not enough information is currently available regarding gender-based and socioeconomic differences in DOAC prescription in our geographical area.

Studying gender-based and socioeconomic differences in health care is essential for identifying modifiable causes of inequality and developing solutions to guarantee equity and quality in health care. The aim of this study is to assess gender-based and socioeconomic differences in the prescription of DOACs in people with non-valvular atrial fibrillation seen in Primary Care in Catalonia (Spain).

## 2. Materials and Methods

### 2.1. Study Design and Population

As part of the Fantas-tic study in Catalonia, we used a cross-sectional design and real-world data from patients seen in Primary Care centers (PCCs) managed by the Catalan Health Institute (ICS). The 287 PCCs included employ 3384 physicians and are responsible for the care of an estimated 5,564,292 people, about 80% of the Catalan population. All registered patients diagnosed with non-valvular atrial fibrillation and treated with oral anticoagulants in 2018 were included. 

Data were drawn from the SIDIAP database (Information System for Research in Primary Care), a representative population-based database in Catalonia that collects anonymized clinical information from different data sources: (a) electronic health records from ICS Primary Care, including sociodemographic characteristics, registered diagnoses coded according to the International Classification of Diseases, 10th revision (ICD-10) [17], general practitioner prescriptions and clinical parameters; (b) laboratory data; and (c) prescription data from the Catalan Health System community pharmacies, based on the Anatomical Therapeutic Chemical (ATC) Classification System codes [18]. 

A total of 97,350 registered patients with a diagnosis of atrial fibrillation from 12 months prior to the study were identified from the SIDIAP database, and all those who had an active prescription for oral anticoagulants on 1 January 2018 were included. All authorized anticoagulant treatments with VKAs (acenocoumarol and warfarin) and DOACs (dabigatran, rivaroxaban, apixaban and edoxaban) in Spain in 2016 were included in the study. Drug data based on ATC codes were collected [18]. 

### 2.2. Inclusion and Exclusion Criteria 

We included patients under treatment with oral anticoagulants and followed in PCCs who had been diagnosed with non-valvular atrial fibrillation at least one year before the study date (1 January 2018) and had at least six controls of the international normalized ratio (INR) over the previous 12 months. This restriction was aimed at minimizing INR variability at the start of the treatment and avoiding the effect of temporary withdrawal of VKAs in patients with good INR control. Patients were considered to have been exposed to anticoagulation if they had been prescribed anticoagulants (acenocoumarol, warfarin, dabigatran, rivaroxaban, apixaban or edoxaban) for at least two months before the start of the study. The anticoagulant medication included in the study was the one which had been started the closest to the study date.

We excluded patients with no oral anticoagulant therapy, patients whose treatment was monitored in hospital, those with valvular atrial fibrillation (mitral stenosis or with a mechanical prosthetic valve), pregnant women, and patients whose anticoagulant treatment at the beginning of the study could not be ascertained.

### 2.3. Study Variables 

*Main variable.* Type of oral anticoagulant prescribed: VKAs (acenocoumarol or warfarin) versus DOACs (dabigatran, rivaroxaban, apixaban or edoxaban), based on ATC classification system codes [18].

*Secondary variables.* Sociodemographic variables related to patients (gender, age) and the socioeconomic deprivation degree of the PCC geographical area. To measure deprivation, we followed the classification used by the Catalan Health Institute, which uses the MEDEA index [8] to rate urban PCCs according to the deprivation level of each PCC area (the census tract corresponding to the PCC area), which is updated when the census is updated, every 10 years (we used the results calculated for 2018, the period of study). The MEDEA instrument classifies urban areas on a scale from MEDEA 1 (low deprivation) to MEDEA 5 (high deprivation). As a composite deprivation index, it assesses barriers to accessing employment, education, culture and social development at a level that is considered acceptable to the society or surrounding region, and it is composed of subindicators for employment and education [5]. As the MEDEA was initially designed for urban areas, based on an analysis of five large Spanish cities [8], rural PCCs were not included in the classification. In our study, rural PCCs were grouped into a separate category and defined as centers serving a population of less than 10,000 inhabitants and with a population density of less than 150 inhabitants/km^2^ [19].

Other secondary variables included clinical variables: time since diagnosis of atrial fibrillation; health care setting where oral anticoagulants were prescribed (Primary Care or hospital); history of cardiovascular disease; intracranial bleeding; comorbidities; risk factors for bleeding; risk scores based on participants’ real-world data (CHA_2_DS_2_-VASC for stroke risk and HAS-BLED for bleeding risk); patients attending outside the PCC (home care or institutionalized care); and teaching PCC. Comorbidities were classified according to the ICD-10 [17].

### 2.4. Statistical Analysis 

Data cleaning was performed by verifying minimum and maximum values and by analyzing missing data. 

The treatment variable was classified as VKA or DOAC. Once the database was cleaned, a descriptive analysis was undertaken. Categorical variables were expressed as absolute and relative frequencies and continuous variables as median (interquartile range, IQR). Included patients were described according to their treatment and other characteristics, and they were compared by using the two proportion Z-test for categorical variables and the non-parametric Mann–Whitney U test for continuous variables.

To test the association between the type of treatment and the rest of the variables, and to study the factors related to the prescription of DOACs, we calculated the adjusted odds ratio (ORadj) using a multivariable logistic regression model. The statistical analysis was performed using Microsoft Office Excel 2013 (Redmond, Washington, USA) and SPSS version 20.0 software (Armonk, New York, NY, USA).

## 3. Results

The included population comprised 60,978 patients with non-valvular atrial fibrillation on anticoagulant therapy: 41,430 (68%) taking VKAs and 19,548 (32%) DOACs.

Regarding gender differences in the type of oral anticoagulant prescribed, a higher proportion of women were prescribed DOACs than men (Table 1). Of the patients receiving DOACs, 50.1% were women.

**Figure 1:**
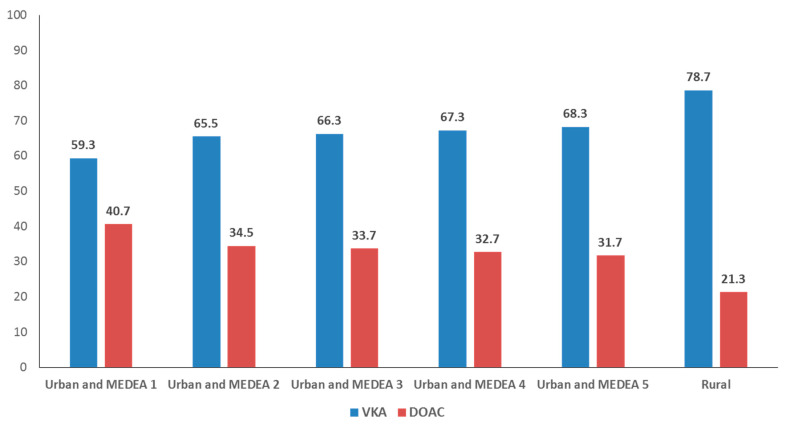
Proportional distribution of prescriptions for vitamin K antagonists (VKA) versus direct oral coagulants (DOAC) according to the deprivation index of urban Primary Care centers (high MEDEA score = more deprivation) or rurality.

There were also differences according to the level of socioeconomic deprivation; patients whose PCC area was classified as the least deprived (MEDEA 1) were more likely to be prescribed DOACs (12.8% VKA versus 18.6% DOAC; *p* < 0.001). The smallest proportion of patients receiving DOACs were those who attended rural PCCs (22.6% VKA versus 13.0% DOAC; *p* < 0.001). Figure 1 presents the proportional distribution of VKA and DOAC prescriptions by PCC category (MEDEA index 1 to 5, rural PCCs). In the centers classified as MEDEA 1, the difference in the prescription between VKAs and DOACs is smaller (59.3% VKA versus 40.7% DOAC) than in rural PCCs (78.7% VKA versus 21.3% DOAC).

Patients with a history of cardiovascular diseases, cerebrovascular diseases and gastrointestinal bleeding were prescribed DOACs more frequently than VKAs. On the other hand, DOAC prescriptions were more frequent in people with a score of less than 2 on the CHA_2_DS_2_-VASC tool for assessing risk of stroke and in patients who attended outside the PCC premises (home care and institutional care) (Table 1).

According to the results of the logistic regression, the variables associated with differences in prescription of DOACs versus VKAs are: gender, socioeconomic deprivation and rurality. The logistic regression showed that being a woman was associated with DOAC prescription (Table 2). As the level of socioeconomic deprivation rose, the odds of being prescribed DOACs decreased (taking MEDEA 1 levels as the reference). Thus, the highest level of deprivation, MEDEA 5, showed the lowest odds of DOAC prescription among urban areas (ORadj 0.58; *p* < 0.001). However, a rural PCC location was the most important factor associated with lower DOAC prescription rates (ORadj 0.34, *p* < 0.001; Table 2).

Advanced age, arterial hypertension, renal insufficiency and longer time since diagnosis of atrial fibrillation were associated with a lower frequency of DOAC prescription (Table 2). In contrast, a history of ischemic cardiopathy, peripheral artery disease, heart failure, gastrointestinal bleeding and cerebrovascular events were associated with higher odds of being prescribed a DOAC. 

**Figure 2:**
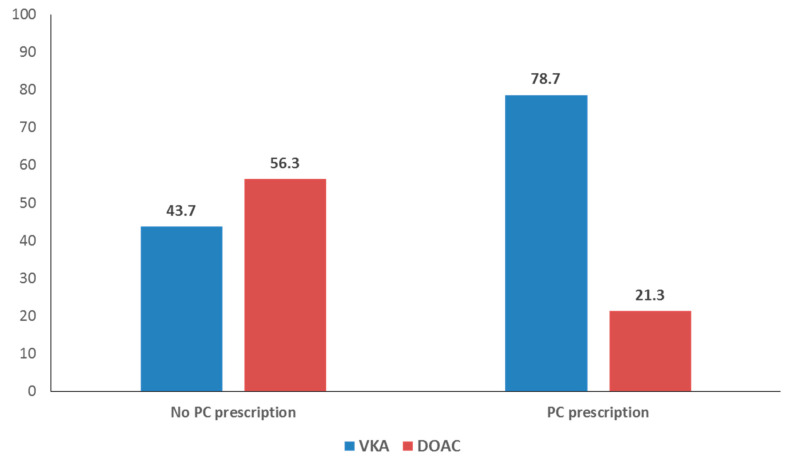
Proportional distribution of prescriptions for vitamin K antagonists (VKA) versus direct oral coagulants (DOAC) according to whether medication was prescribed inside or outside of a Primary Care center.

Patients who attended outside the PCC, whether at home or in an institution, were more likely to be prescribed a DOAC. On the other hand, receiving care in a teaching PCC or receiving the anticoagulant prescription in a PCC was associated with lower DOAC prescription rates. Figure 2 shows the proportional distribution of prescriptions (DOAC versus VKA) according to whether the patient received the prescription in or outside a PCC. Within PCCs, 21.3% of the prescriptions for oral anticoagulants are DOACs, while outside the centers, this figure stands at 56.3%.

## 4. Discussion

This study, analyzing real-world data, identified differences in the prescription of different types of oral anticoagulants (VKA versus DOAC) based on the characteristics of patients and the PCC area. The main factors associated with the type of drug prescribed were gender, socioeconomic deprivation of the urban area and rurality.

Being a woman was associated with more frequent prescriptions for DOACs, after adjusting for the rest of the variables. Lower DOAC prescription was associated with both socioeconomic deprivation and rurality.

The statistical differences detected with regard to gender, deprivation and rurality could reflect inequality in the prescription patterns of oral anticoagulants if these differences are avoidable. If the inequality is unfair, it would represent an example of inequity, an ethical concept that considers inequality on the basis of a values system. The central criterion to define inequality as inequitable is fairness, where inequity is defined as an unfair inequality [20].

Socioeconomic determinants such as education, employment status, income, gender and ethnicity have a clear influence on an individual’s health. The lower socioeconomic status a person has, the higher their risk for poor health [20]. Health inequities are systematic differences in the health status of different population groups. These inequities have a high social and economic cost for both individuals and the society as a whole [20]. In Sweden, socioeconomic inequalities in the prescription of oral anticoagulation for preventive treatment after stroke were based on age, income, education and country of birth [11]. In Denmark, patients with atrial fibrillation had a lower chance to being initiated with oral anticoagulation therapy when they had low income, low education and were living alone. Inequality reduced when more detailed new guidelines were published in 2011 [12]. Following clinical guideline recommendations improves adequacy and reduces inequality.

Oral anticoagulants (VKAs and DOACs) are medicines of proven efficacy and effectiveness in preventing thromboembolic events in non-valvular atrial fibrillation. DOACs are considerably more expensive than VKAs, but they are also more practical to use because patients on DOACs do not require close follow-up, as they do on VKAs. In some circumstances, for instance in patients with a history of intracranial bleeding, DOACs have also demonstrated more safety [1]. The differences in cost, convenience and even in therapeutic advantages, could be the cause of inequities in prescription patterns that are related to the differences detected in socioeconomic deprivation.

The association between being a woman and being prescribed DOACs shows that women are more likely to receive these drugs than men, even after adjusting for age, medical history, MEDEA index and prescription setting. Other factors not analyzed in this study could also have had an impact, such as polypharmacy (more prevalent in women) or the lower time in therapeutic range observed in women [21]. Gender inequality in the prescription of analgesics and antidepressants is well documented in our country, and the observed differences cannot be fully explained by clinical factors [22]. Specifically, the prescription of analgesics in Spain is more frequent in women than in men, especially in people with a low educational and socioeconomic level [22]. On the other hand, women, especially those with a low socioeconomic status, are more likely to be diagnosed with depression and prescribed antidepressants and other psychotropics. These differences cannot be attributed to a higher frequency of symptoms of depression or visits to Primary Care [7]. 

Our results also indicate that a younger age is associated with higher prescription of DOACs, even after adjusting for other included variables. Some factors that we did not study may have influenced this result, for example the loss of labor productivity due to follow-up appointments, patient preferences, purchasing power, and co-payments for the medication. However, younger age also tends to indicate less severe pathology, and in turn a lower probability of meeting the current medication appropriateness criteria for DOACs [23,24]. Lower age is also associated with a lower CHA_2_DS_2_-VASC score (<2), in which case treatment with oral anticoagulants would not be appropriate [23].

The influence of socioeconomic factors on inequalities in DOAC prescriptions and the relation to its high cost has been studied in different countries. In line with our results, the literature shows that low socioeconomic status is associated with lower use of DOACs in different geographic areas and contexts [3,25]. In Denmark, increasing inequality was observed regarding high-cost drugs, such as DOAC, for the treatment of atrial fibrillation [12]. The way that medications are financed and the type of health system seems to play a relevant role in prescription patterns [3].

The substantial difference in the cost of treatment can hinder the prescription of DOACs in areas affected by greater socioeconomic deprivation, as observed in our study. Rural residence is one of the factors that is most closely associated with lower prescription rates of DOACs. However, the greater convenience associated with following patients treated with DOACs versus VKAs makes the former a more functional treatment for patients who have difficulties in accessing health centers. There is some controversy around whether the higher cost of DOACs compared to VKAs is offset by the lower need for follow-up, making the total healthcare expenditure comparable between the two treatments [26]. Studies being carried out in our context will provide evidence of the cost-effectiveness of both classes of medication [27]. The treatment strategy must consider a holistic, integrated assessment of the patient within the framework of current guidelines.

The MEDEA index used in our study has a limitation in terms of its interpretation, as this index of socioeconomic deprivations is linked to the health center and therefore reflects the index of the census tract corresponding to the basic health area. This means that the patients seen in each PCC may have a different level of deprivation than the area in which they live; although, in general, evidence shows that in population terms, the socioeconomic situation of the census tract is related to mortality in the resident population [19]. Furthermore, potential physician conflicts of interest with pharmaceutical companies cannot always be discarded, which could influence prescription decisions. In other cases, physicians could consider the patient affluent and could prescribe DOAC, which although they are more expensive are more comfortable.

This study opens the door to new studies that can help establish the socioeconomic determinants of inequalities in DOAC prescriptions and assess whether these are avoidable, for the ultimate purpose of achieving fairer and more equitable prescription patterns. The inequality observed in prescriptions of oral anticoagulants should be minimized by following current atrial fibrillation management guidelines and addressing modifiable determinants in the pursuit of healthcare equity, better health and social justice.

## 5. Conclusions

This study reveals differences in prescription patterns for oral anticoagulants, specifically in relation to DOACs. Being a woman was associated with higher prescription rates for DOACs, while lower prescription rates were seen in socioeconomically deprived and rural areas. This means that DOACs, co-financed drugs, are prescribed more to women than to men (young), and more to patients with high socioeconomic status than those with low socioeconomic status. These differences could not be explained by the adequacy factors included in the recommendations of the current guidelines for the treatment of atrial fibrillation. Thus, reflecting inadequacy in the treatment. Future studies should identify modifiable factors associated with the inequalities detected. Current atrial fibrillation clinical management guidelines needs to be followed in order to minimize the inequality. 

## Figures and Tables

**Table 1:** Description of patient population according to class of oral anticoagulants prescribed.

Variables	Vitamin K Antagonists		Direct Oral Anticoagulants		*p* Value
	*n* *	%	*n* *	%	
***Primary care center characteristics***					
Classification by rurality and socioeconomic deprivation (urban settings)					
MEDEA 1	5296	12.8	3630	18.6	<0.001
MEDEA 2	6142	14.8	3230	16.5	<0.001
MEDEA 3	6457	15.6	3285	16.8	<0.001
MEDEA 4	6636	16.0	3224	16.5	0.14
MEDEA 5	5909	14.3	2747	14.1	0.49
Rural	9350	22.6	2532	13.0	<0.001
Missing	1640	4.0	900	4.6	<0.001
Prescription in Primary Care	33,263	80.3	9009	46.1	<0.001
Care setting outside PCC					
Home care	4926	11.9	2906	14.9	<0.001
Institutional care	1646	4.0	1016	5.2	<0.001
Teaching center					
Yes	11,305	27.3	5117	26.2	0.004
Missing	176	0.4	124	0.6	0.001
***Patient characteristics***					
Total	41,430	67.9	19,548	32.1	<0.001
Women	20,285	49.0	9800	50.1	0.007
Men	21,145	51.0	9748	49.9	0.007
Age group					
<60 years	935	2.3	1031	5.3	<0.001
60–69 years	4682	11.3	2893	14.8	<0.001
70–79 years	13,654	33.0	6061	31.0	<0.001
≥80 years	22,159	53.5	9563	48.9	<0.001
Age in years (median, IQR)	80.0	11.0	79.0	14.0	<0.001
Years since diagnosis of atrial fibrillation (median, IQR)	4.0	6.0	3.0	5.0	<0.001
History of cardiovascular disease					
Peripheral artery disease	2704	6.5	1489	7.6	<0.001
Ischemic cardiopathy	7514	18.1	4108	21.0	<0.001
Aortic atheromatosis	400	1.0	234	1.2	0.009
Cerebrovascular event					
Ischemic stroke	6436	15.5	4693	24.0	<0.001
Unspecified cardiovascular accident	559	1.3	392	2.0	<0.001
Intracranial bleeding	364	0.9	462	2.4	<0.001
Comorbidities					
Diabetes mellitus	13,587	32.8	6474	33.1	0.43
Hypertension	33,360	80.5	15187	77.7	<0.001
Heart failure	10,846	26.2	5253	26.9	0.070
Renal insufficiency	12,340	29.8	5281	27.0	<0.001
Bleeding risk factors					
Alcohol consumption	1727	4.2	843	4.3	0.41
Brain aneurism	28	0.1	35	0.2	<0.001
Portal hypertension	80	0.2	24	0.1	0.050
Hepatic insufficiency	263	0.6	118	0.6	0.65
Hereditary hemorrhagic telangiectasia	2	0.0	2	0.0	0.44
Aneurism and aortic dissection	614	1.5	322	1.6	0.12
Intestinal angiodysplasia	92	0.2	85	0.4	<0.001
Gastrointestinal bleeding	3286	7.9	1852	9.5	<0.001
Bleeding other than gastrointestinal or intracranial	569	1.4	269	1.4	0.98
Stroke risk (CHA_2_DS_2_-VASC)					
0	404	1.0	499	2.6	<0.001
1	2107	5.1	1613	8.3	<0.001
2	7251	17.5	3472	17.8	0.43
3	14,681	35.4	5820	29.8	<0.001
≥4	16,987	41.0	8144	41.7	0.12
Bleeding risk (HAS-BLED)					
0	953	2.3	1063	5.4	<0.001
1	14,563	35.2	7582	38.8	<0.001
2	15,112	36.5	6528	33.4	<0.001
3	7783	18.8	3164	16.2	<0.001
≥4	3019	7.3	1211	6.2	<0.001

* Unless otherwise noted. IQR: interquartile range; PCC: Primary Care center.

**Table 2:** Association between the prescription of direct anticoagulants versus vitamin K antagonists according to patient- and center-related characteristics. Multivariable logistic regression.

	OR_adj_	95% CI	*p*
***Primary care center characteristics***			
Classification by rurality and socioeconomic deprivation (urban settings)			
MEDEA 1	1		
MEDEA 2	0.69	(0.65–0.74)	<0.001
MEDEA 3	0.64	(0.60–0.68)	<0.001
MEDEA 4	0.61	(0.57–0.65)	<0.001
MEDEA 5	0.58	(0.54–0.62)	<0.001
Rural	0.34	(0.32–0.36)	<0.001
Care setting outside PCC			
Home care	1.30	(1.22–1.37)	<0.001
Institutional care	1.20	(1.09–1.32)	<0.001
Prescription in Primary Care			
No	1		
Yes	0.22	(0.21–0.23)	<0.001
Teaching center			
No	1		
Yes	0.88	(0.85–0.93)	<0.001
***Patient characteristics***			
Gender			
Men	1		
Women	1.12	(1.08–1.16)	<0.001
Age group			
<60	1		
60–69	0.59	(0.52–0.66)	<0.001
70–79	0.43	(0.38–0.47)	<0.001
≥80	0.41	(0.37–0.45)	<0.001
Years since diagnosis of atrial fibrillation	0.98	(0.97–0.98)	<0.001
History of cardiovascular disease			
Peripheral artery disease	1.14	(1.05–1.22)	<0.001
Ischemic cardiopathy	1.15	(1.09–1.20)	<0.001
Aortic atheromatosis	1.11	(0.93–1.33)	0.26
Cerebrovascular event			
Ischemic stroke	1.64	(1.56–1.72)	<0.001
Unspecified cardiovascular accident	1.23	(1.06–1.43)	0.005
Intracranial bleeding	2.70	(2.33–3.13)	<0.001
Comorbidities			
Diabetes mellitus	1.01	(0.97–1.05)	0.62
Hypertension	0.88	(0.83–0.92)	<0.001
Heart failure	1.06	(1.02–1.11)	0.004
Renal insufficiency	0.88	(0.85–0.93)	<0.001
Bleeding risk factors			
Alcohol consumption	0.92	(0.83–1.01)	0.094
Brain aneurism	1.18	(0.65–2.13)	0.59
Portal hypertension	0.61	(0.37–1.02)	0.059
Hepatic insufficiency	0.88	(0.68–1.12)	0.29
Hereditary hemorrhagic telangiectasia	1.27	(0.14–11.11)	0.83
Aneurism and aortic dissection	1.10	(0.94–1.30)	0.21
Intestinal angiodysplasia	1.79	(1.30–2.50)	<0.001
Gastrointestinal bleeding	1.22	(1.15–1.32)	<0.001
Bleeding other than gastrointestinal or intracranial	0.92	(0.78–1.09)	0.30

ORadj: Adjusted odds ratio; CI: confidence interval; PCC: Primary Care center.

## Data Availability

The data that support the findings of this study were obtained from SIDIAP database (Information System for Research in Primary Care). This database is representative of the Catalan population. Restrictions apply to the availability of these data, which were used under license for this study. The authors are not authorized to share the data.
